# Case Report: Lung cancer with rare cardiac and other multiple metastases

**DOI:** 10.3389/fcvm.2024.1417906

**Published:** 2024-09-12

**Authors:** Li Chen, Jing Zhang, Chunquan Zhang

**Affiliations:** ^1^ Department of Ultrasound, The Second Affiliated Hospital of Nanchang University, Nanchang, China; ^2^ Jiangxi Medical College of Nanchang University, Nanchang, China

**Keywords:** left atrial tumors, metastasiscase, case report, microenvironment, diagnostic

## Abstract

Metastasis to the left atrium is exceptionally uncommon, occurring at a rate of only 3.1%. The clinical manifestations of lung cancer metastasizing to the heart can vary widely. They range from paraneoplastic syndrome, dyspnea, and ST-segment elevation on an electrocardiogram to no clinically significant symptoms. Diverging from typical metastatic patterns observed in lung cancer, this case report presents a detailed description, from the perspective of the microenvironment, of a rare instance where lung cancer metastasized to the mediastinal lymph nodes, adrenal glands, brain, and notably, the left atrium, in a non-smoking female patient.

## 1 Introduction

Globally, lung cancer stands as the foremost cause of cancer-related mortality among both men and women, boasting an exceedingly high mortality rate (1). The global increase in lung cancer incidence is primarily driven by male cases, with 85%–90% of these cases closely associated with smoking (2). However, recent studies have revealed a rising incidence of lung cancer among non-smoking women, and it is anticipated that by 2,045, the lung cancer mortality rate in women will surpass that in men (3). It is classified into two main categories: small-cell lung cancer and non-small-cell lung cancer. The predominant sites of metastasis for non-small-cell lung cancer, in descending order, include bone, lung, brain, adrenal gland, liver, and extrathoracic lymph nodes (4). Among these, the likelihood of metastasis to the brain, adrenal gland, and extrathoracic lymph nodes was 28.4%, 16.7%, and 9.5%, respectively (4). Nevertheless, metastasis to the heart is deemed exceptionally rare, with only 3.1% of 4,668 lung cancer patients developing such metastasis in one study (5). This case report is the first to detail, from the perspective of the microenvironment, a female lung cancer case with multiple rare site metastases–including the heart, mediastinal lymph nodes, adrenal glands, and brain–presenting with no significant clinical symptoms other than weight loss.

## 2 Case report

**Figure 1:**
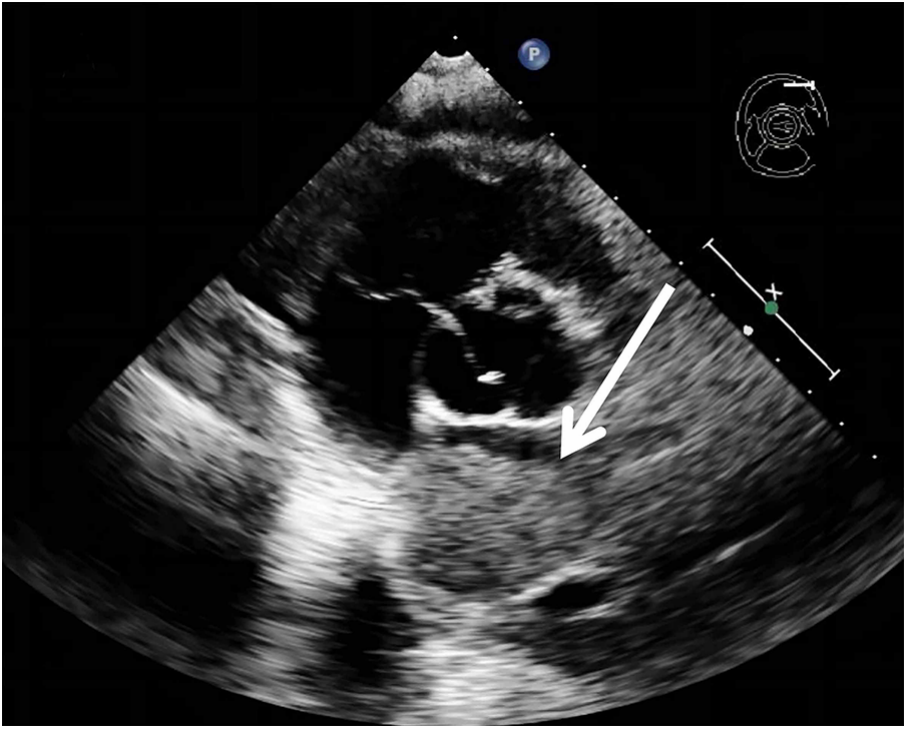
A metastatic hypoechoic mass, measuring 5.1 × 3.4 cm, was present in the left atrium, closely adjacent to the right pulmonary vein.

**Figure 2:**
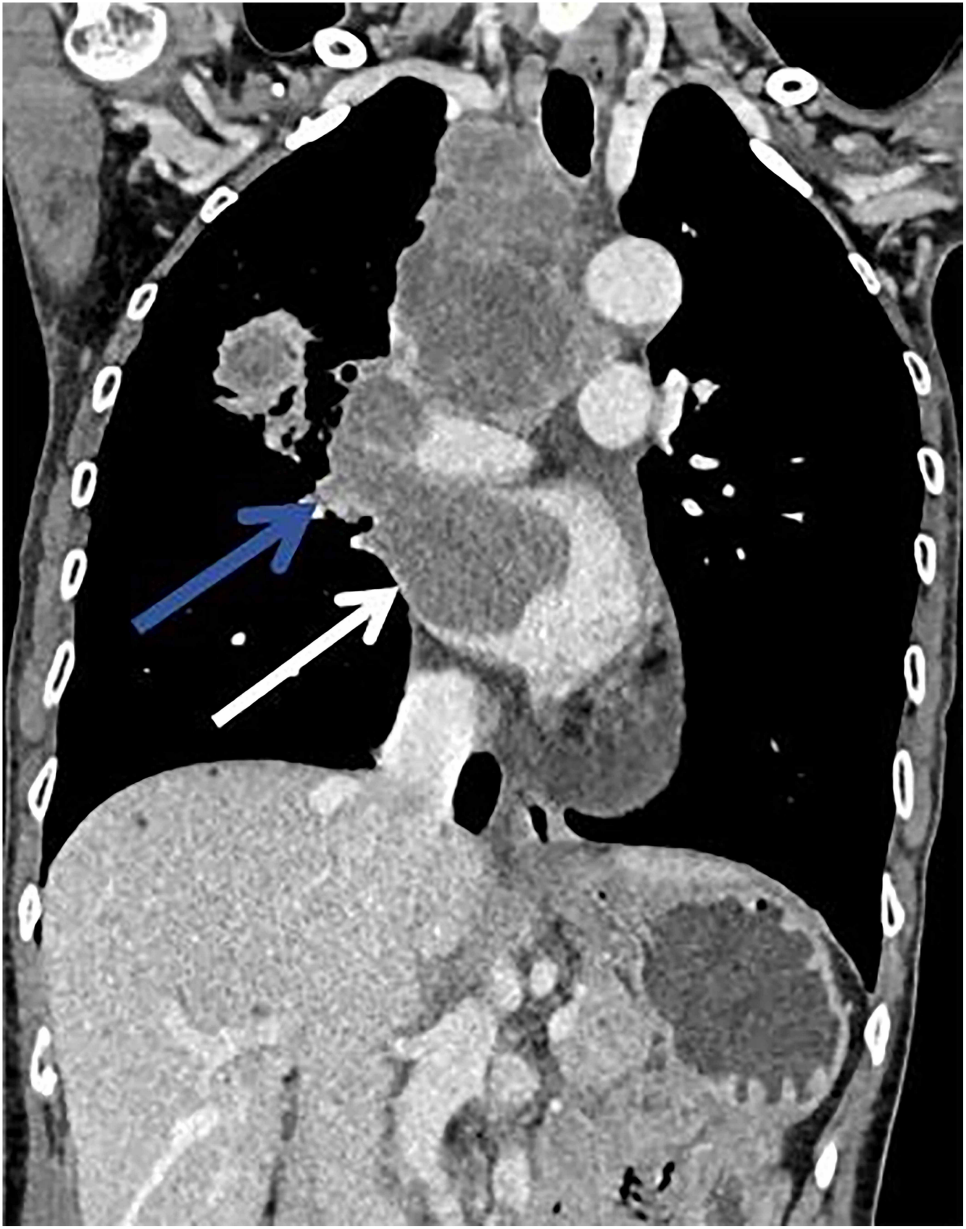
There was a mild enhancement filling defect noted in the right upper pulmonary vein (blue arrow) and the left atrium (white arrow).

**Figure 3:**
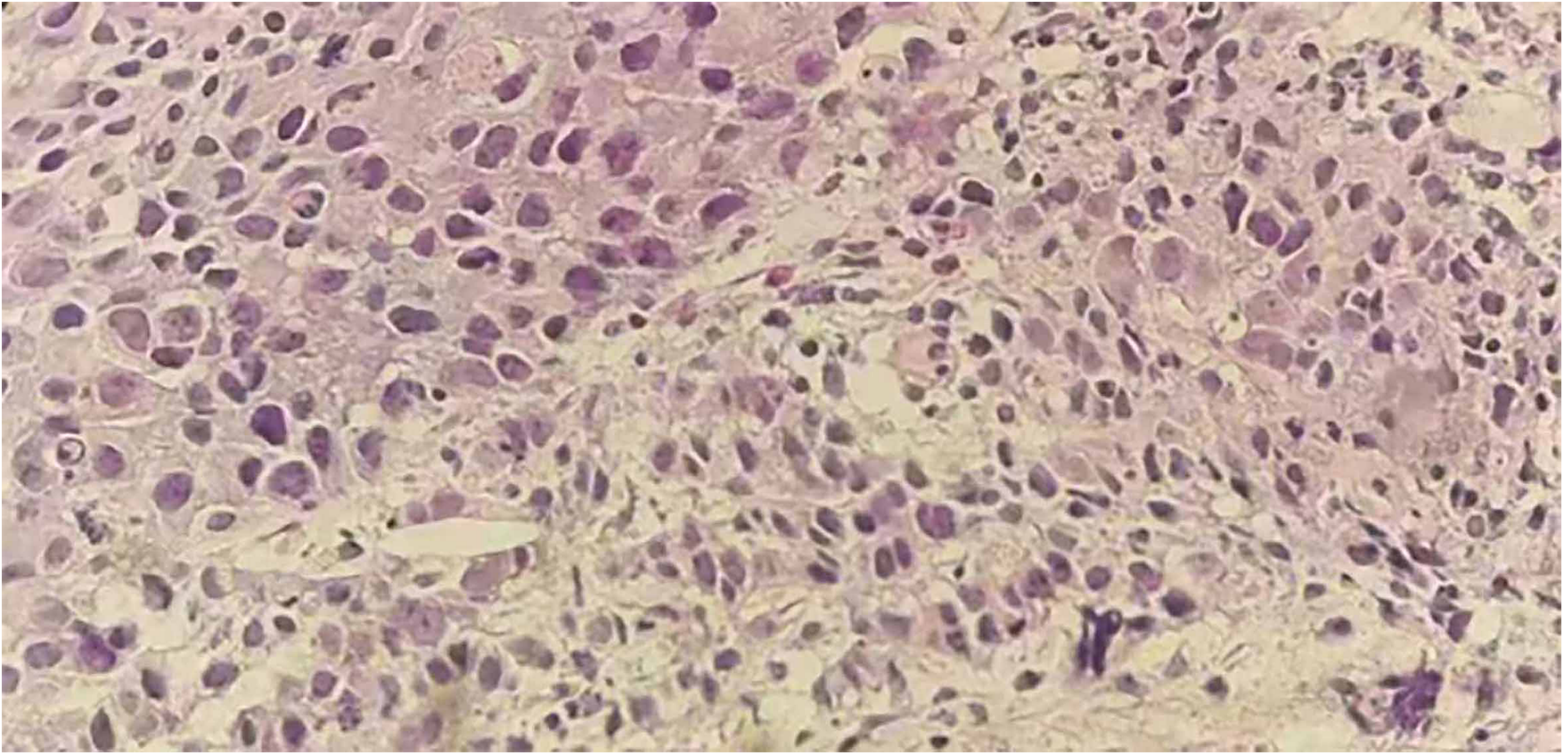
Hematoxylin and eosin (H&E) staining on the lung tissue sections reveals nest-like formations of cancer cells, invasive growth patterns, enlarged and deeply stained nuclei, a high nucleus-to-cytoplasm ratio, pronounced atypia of the cancer cells, and stromal fibrosis.

A 62-year-old non-smoking female presented to our hospital with kidney stones and underwent a comprehensive full-body examination. She self-reported being previously healthy and not experiencing any symptoms, except for a significant recent weight loss. During the physical examination, the patient appeared to be in good overall condition, with a blood pressure reading of 92/51 mmHg, a heart rate of 81 beats per min, a respiratory rate of 19 breaths per min, and no murmurs detected upon cardiopulmonary auscultation. The chest CT scan revealed lung cancer in the upper lobe of the right lung, which was further complicated by hilar and mediastinal lymph node metastasis. The lymphatic vessels surrounding the tumor appeared thickened and nodulated. Transthoracic echocardiography and organ acoustic imaging identified a 5.1 × 3.4 cm metastatic hypoechoic mass in the left atrium, closely associated with the right pulmonary vein (Figure 1). She underwent additional CT contrast imaging of the neck, chest, and upper abdomen, which confirmed the presence of a mass in the left atrium connected to the right upper pulmonary vein (Figure 2). Furthermore, a metastatic nodule was detected in the right adrenal gland. Simultaneously, a contrast-enhanced MRI of the head revealed a circular enhancing nodule located centrally within an oval lesion on the left side, indicative of skull metastasis. To elucidate the pathology, the patient ultimately opted for lung biopsy and immunohistochemical examination, which confirmed the diagnosis of non-small-cell lung cancer (NSCLC). Microscopic examination revealed cancer cells arranged in nests and clusters, demonstrating infiltrative growth, notable heterogeneity among the cancer cells, and proliferation of interstitial fibrous tissue (Figure 3). Following antigen retrieval, blocking, antibody staining, and color development steps in immunohistochemistry, the results indicate that the cancer cells exhibited positivity for CK, focal positivity for CK7, TTF1 (localized), and PD-L1 22C3 TPS (localized), with a Ki-67 proliferation index of approximately 20%. The cells tested negative for NapsinA, P40, P63, CK5/6, and ALK-D5F3. Special stains indicated negative staining for mucus carmine and positive staining for individual cells of AB-PAS.

However, the tumor had metastasized to multiple distant sites, progressing to stage IVB, thus eliminating the possibility of surgical intervention. To identify a more appropriate chemotherapy regimen, the patient underwent genetic testing, revealing a fourth exon TP53 frameshift mutation (abundance 48.6%), a KMT2A exon 22 mutation (abundance 25.95%), and an MSH2 exon 16 mutation (abundance 2.6%). The primary method for genetic testing involves fixing, dehydrating, and sectioning the tumor tissue samples obtained from biopsy, followed by DNA extraction and whole-exome sequencing. After careful deliberation, the physician suggested an immunotherapy protocol involving a combination of carboplatin and pemetrexed chemotherapy along with tislelizumab. During the therapy, the patient did not report any significant discomfort. Although the degree of tumor shrinkage still requires further observation, the patient's overall condition remains stable. Specifically, the performance status score is 1, the numerical rating scale is 1, body weight has remained stable, and no significant adverse reactions or complications have emerged. The patient has exhibited a commendable tolerance to this treatment regimen. However, due to the patient's financial constraints and psychological pressures, the patient ultimately decided to discontinue treatment. Specifically, the substantial therapy costs imposed severe stress on her financial situation. Additionally, the patient experienced significant psychological burdens during the therapy, perceiving its prolonged nature and impact on her quality of life, which influenced her decision to cease further treatment. Clinical events have been listed according to the timeline (Table 1).

**Table 1:** Based on the timeline, clinical events are listed below.

Time	Evens
April 10, 2023	A transthoracic echocardiogram detected a hypoechoic mass located at the base of the left atrium, measuring approximately 5.1 × 3.4 cm, exhibiting indistinct demarcation from the right pulmonary vein and demonstrating minimal motion throughout the cardiac cycle.
April 11, 2023	Through visceral acoustic imaging, it was confirmed that the left atrial metastasis originated from the right pulmonary vein. A thoracic CT scan diagnosis suggested lung cancer with multiple metastases.
April 13, 2023	Under the guidance of CT laser localization, a lung biopsy was successfully performed.
April 14, 2023	Enhanced CT scans of the neck, chest, and upper abdomen revealed lung cancer with tumor emboli formation in the right upper pulmonary vein and the left atrium, along with metastases to extrathoracic lymph nodes, adrenal glands, and brain. Pathological examination confirmed infiltrating carcinoma in the right lung. The patient received symptomatic treatment with heparin anticoagulation.
April 19, 2023	Immunohistochemistry indicated non-small-cell lung cancer in the right lung.
May 8, 2023	The patient underwent chemotherapy with a combination of carboplatin and pemetrexed chemotherapy along with the tislelizumab and received supportive care, including gastric protection and antiemetics.
May 15, 2023	After this treatment course, the patient's general condition was satisfactory. However, due to personal reasons, she ultimately opted to discontinue further treatment.

## 3 Discussion

We report a case of a female non-smoker with lung cancer, presenting with metastases to multiple sites, including the left atrium and extrathoracic lymph nodes. Traditionally, the occurrence of lung cancer has been closely associated with male smokers, and the clinical manifestations of left atrial metastases can vary widely, ranging from paraneoplastic syndromes, dyspnea, ST-segment elevation on electrocardiograms, hemoptysis, and weight loss, to the absence of noticeable clinical symptoms (6–9). In contrast to other patients with lung cancer metastasizing to the left atrium, this case involves a female non-smoker who, aside from weight loss, exhibits no other clinical symptoms, which is exceedingly rare (Table 2). In recent years, the incidence of lung cancer among female non-smokers has been rising, potentially linked to exposure to smoke from charcoal, heating, or cooking (1). The increasing incidence of lung cancer among female non-smokers presents a new challenge to current screening programs and strategies, necessitating further adjustments to accommodate this emerging risk group. Remarkably, the patient's circulating tumor cells disseminated directly to the left atrium via the pulmonary veins, an exceedingly rare occurrence (15). Cardiac tumors are categorized into primary cardiac tumors and metastatic cardiac tumors, with metastatic cardiac tumors being 20–30 times more prevalent than primary cardiac tumors (16). However, the reported incidence of left atrial metastatic tumors is only 3.1% (5). The microenvironment, an intricately structured ecosystem consisting of diverse immune cells, cellular stroma, and vascular cells, is widely recognized to play a crucial role in the progression of metastatic tumors (17, 18). It facilitates the dissemination of tumors to distant sites through mechanisms involving inflammatory cytokines, and the Wnt/T-catenin pathway, among others (19). Thus, it plays a central role in tumorigenesis and metastasis (20). In this report, lung cancer cells breached the vascular basement membrane and endothelial barrier to metastasize to the left atrium via the pulmonary vein, a process potentially linked to CC-chemokine receptor 2 (CCR2) signaling by monocytes. CCR2 signaling monocytes transition into mobile tumor-associated macrophages and are converted into the chemokine receptor 4 -expressing macrophages upon exposure to transforming growth factor *β*. CXC-chemokine ligand 12 (CXCL12), secreted by stromal fibroblasts near blood vessels, triggers migrating macrophages and cancer cells to aggregate toward blood vessels (21). Subsequently, the aggregated migrating macrophages transform into perivascular macrophages and enhance vascular permeability by inducing vascular endothelial growth factor A signaling. This results in local disruption of vascular junctions, thereby facilitating the invasion of cancer cells into the vasculature to become circulating tumor cells (22). Circulating tumor cells directly or indirectly interact with various cell types such as blood cells, endothelial cells, and cancer-associated fibroblasts. They manipulate the cellular functions of surrounding normal cells, facilitating the extravasation of circulating tumor cells into the left atrium and leading to the development of left atrial metastases (23). It is also posited that tumor development is closely associated with endoplasmic reticulum (ER) stress (24). In various tumors, the combination of carcinogenesis, transcriptional alterations, and metabolic abnormalities leads to a detrimental microenvironment that disrupts endoplasmic reticulum homeostasis, thereby triggering persistent ER stress. This stress state influences the pro-tumorigenic characteristics of cancer cells and dynamically alters the functions of innate and adaptive immune cells. Abnormal activation of ER stress sensors and their downstream signaling pathways constitutes a critical regulatory factor in tumor growth and metastasis. Research also suggests that inflammation within the tumor microenvironment, particularly that driven by tumor-associated macrophages, is commonly regarded as a significant characteristic of tumors (25). High infiltration of tumor-associated macrophages is often closely associated with poor prognosis in various cancers, such as bladder cancer (26). However, other studies have indicated that in certain instances, the infiltration of tumor-associated macrophages may be associated with a more favorable prognosis (27). This may be related to certain factors within the tumor. In studies of NSCLC, the relationship between macrophages and patient survival has been investigated (28). The research suggests that macrophages within tumor islets may play a role in either promoting tumor growth or exhibiting anti-tumor effects. In contrast, macrophages in the tumor stroma could be associated with tumor progression and adverse prognosis. This indicates that tumor-associated macrophages may hold significant roles in the development and metastasis of NSCLC. Furthermore, tumor-associated macrophages may exhibit varying functions and prognostic implications across different regions of the tumor microenvironment, potentially offering new insights for personalized treatment and prognostic evaluation.

**Table 2:** A review of cases with lung cancer metastasis to the left atrium.

	Our case	Clarket et al. (10)	Nosrati et al. (11)	Xie et al. (12)	Uygur et al. (13)	Cipriano et al. (14)
Gender	Women	Women	Men	Men	Men	Men
Age	62	38	72	59	68	62
Smoker?	NO	NO	YES	Indeterminate	YES	YES
Primary lesion	NSCLC	NSCLC	NSCLC	NSCLC	undifferentiated round cell malignant neoplasm	NSCLC
Clinical characteristics	Weight loss	Cough, shortness of breath, loss of appetite, weig-ht loss	Hemoptysis, fainting	Progressive dys-pnea and exacerbation of bilateral lower extremity edema.	Difficulty breathing, cough	Dyspnea, dry cough, chest and back pain, and weight loss
Diagnostic methods	x-ray, CT, echo-cardiogram, MRI, organ acoustic imaging	x-ray, CT	CT, echocardiogram	CT, echocardiogram, MRI	CT, echocardiogram	CT, echocardiogram
Treatment strategies	carboplatin and pemetrexed chemotherapy along with tislelizumab	radiotherapy, Paclitaxel combined with carboplatin chemotherapy. Pembrolizumab immunotherapy	radiotherapy, Chemotherapy with Etoposide and Cisplatin	Lobectomy, tumor resection, and partial left a-trial resection	Tumor excision, lobectomy, and postoperative chemotherapy	Resection of the cardiac tumor, lobectomy, followed by adjuvant radiotherapy and chemotherapy
Outcome	Discontinuation of treatment	Reduction in tumor size	Deceased	Survived	Survived	Deceased

Currently, due to the lack of specific clinical manifestations of left atrial metastases, clinical symptoms cannot serve as direct evidence for the diagnosis of cardiac tumors, often leading to late and incidental discoveries. The diagnosis of cardiac tumors relies on pathological biopsy; however, due to the invasive nature of this method, it is frequently not acceptable to patients (29). In this context, imaging assessment of cardiac tumors has become an increasingly crucial approach. Transthoracic echocardiography is the preferred imaging modality for the evaluation of cardiac tumors, aiding in the differentiation of cardiac tumors from other cardiac conditions. Additionally, with advancements in ultrasound technology, transesophageal echocardiography provides a higher-resolution view of the fine structures of the heart and the pulmonary veins. Uygur et al. (13) reported a case of lung cancer metastasizing to the left atrium, initially diagnosed as an atrial myxoma by transthoracic echocardiography, but subsequently confirmed as a tumor metastatic via the left upper pulmonary vein through transesophageal echocardiography. CT scanning provides a clear depiction of the tumor's specific location and its relationship with surrounding tissues, aiding in the identification of other metastatic sites and serving as an initial screening for metastasis. However, CT has limitations such as poor soft tissue contrast, radiation exposure, and image artifacts, which may lead to potential misdiagnoses. Cipriano et al. (14) reported a case of cardiac metastasis in which preoperative head CT did not reveal any brain metastases. However, postoperatively, the patient developed seizures, and a subsequent CT scan identified brain metastases. MRI is the standard imaging modality for detecting brain metastases, and, if necessary, patients should undergo multimodal imaging. Bilani et al. (29) investigated the multimodal assessment of cardiac metastases in patients with NSCLC, presenting a detailed clinical case of one patient. The patient's clinical features, along with 18F-labeled fluorodeoxyglucose positron emission tomography (^18^F-FDG PET) and echocardiography, suggested endocardial metastasis. However, due to the patient's anxiety about the MRI, a further MRI evaluation was declined, and pembrolizumab treatment was chosen instead. Subsequent PET-CT scans, performed two months apart, were negative. Nevertheless, after nine months of treatment, PET-CT revealed a recurrence in the left ventricular area. Ultimately, the patient underwent an MRI, which surprisingly yielded a negative result. The case of Bilani et al. (29) underscores the importance of MRI in the diagnosis of tumor metastases and demonstrates its potential applications in multimodal assessment. This case reinforces the critical role of multimodal imaging in enabling clinicians to accurately ascertain a patient's condition, thereby facilitating the selection of the most suitable treatment plan and ensuring early and effective intervention to minimize the wastage of medical resources. The patient in our case underwent comprehensive evaluations, including echocardiography, contrast-enhanced CT, and MRI, which confirmed the diagnosis of lung cancer and its metastatic lesions. Although the patient exhibited no other clinical symptoms beyond weight loss, which is consistent with current research on cardiac tumors (9), this case warrants special attention compared to cardiac tumors presenting with a range of clinical symptoms. Cases with such subtle clinical symptoms not only have the potential to delay diagnosis and treatment decisions but also underscore the critical importance of emphasizing weight changes and the need for early diagnosis and individualized monitoring in cancer screening.

Rare metastatic lung cancer is generally associated with a poor prognosis. However, with the advent of current highly effective systemic therapies, including immunotherapy, chemotherapy, and radiotherapy, the survival of patients with rare metastatic lung cancer has been significantly extended (30). The study by Niu et al. (31) reveals that a combined approach of systemic therapy (such as chemotherapy and targeted therapy) with local treatments (like tumor resection, radiotherapy, or radiofrequency ablation) can significantly prolong overall survival. This finding underscores the superior benefits of integrated treatment in extending survival, in contrast to relying solely on systemic therapy or supportive care, which yield less favorable outcomes. Jasper et al. (30) further emphasized that this integrated approach not only aids in long-term disease control but also holds the potential for achieving curative outcomes. This indicates that an integrated treatment strategy is particularly crucial for patients with rare metastatic lung cancer, as it not only enhances survival but also offers the possibility of more optimistic therapeutic prospects. Overall, these findings endorse the significance of applying an integrated treatment approach in patients with rare metastatic lung cancer, demonstrating that this method can markedly improve therapeutic outcomes and patient survival rates. Furthermore, it provides compelling evidence for clinical practice, suggesting that healthcare teams should consider the potential advantages of integrated treatment in their treatment plans. Clark et al. (10) achieved tumor reduction in patients with lung cancer accompanied by cardiac metastases through a comprehensive treatment regimen combining radiotherapy, chemotherapy, and immunotherapy. Additionally, Xie et al. (12) and Uygur et al. (13) each reported cases of lung cancer with cardiac metastases where patients survived following surgical resection. These studies challenge the traditional notion that tumors directly extending to the heart are deemed inoperable. This finding suggests that surgical intervention for cardiac metastases may hold genuine therapeutic value, indicating a need to reassess the surgical indications for cardiac metastases and explore related treatment strategies further. In the case presented, the patient experienced widespread metastases, including to the heart. After consulting with experts in cardiology, cardiovascular surgery, and oncology, the patient received a treatment regimen comprising carboplatin and pemetrexed chemotherapy combined with tremelimumab immunotherapy. The patient tolerated this treatment regimen relatively well; however, regrettably, due to economic and psychological pressures, the patient ultimately chose to discontinue further treatment. In this case, a multidisciplinary approach was adopted. With advancements in early cancer detection technologies and effective anticancer therapies, the survival period of lung cancer patients has been significantly extended. However, this also brings challenges related to cardiac issues and elevated cardiovascular risk (32). Cardiovascular disease has been identified as the second leading cause of mortality among patients with NSCLC (33). Particularly for lung cancer patients undergoing cardiotoxic anticancer treatments, a multidisciplinary approach becomes increasingly crucial in their management and care. Baseline cardiovascular risk assessment and evaluation for lung cancer patients should include physical examinations, blood pressure measurements, ECG, lipid profiles, hemoglobin A1c, and smoking status (32). Before initiating anticancer therapy, it is essential to manage cardiovascular risk factors as effectively as possible. During treatment, regular blood pressure monitoring and echocardiographic evaluations should be conducted to detect hypertension and rule out potential cardiomyopathy (34).

Moreover, in this case, metastases to the lymph nodes, adrenal glands, and brain may have the following associations with the microenvironment, respectively. Lymph nodes and lymphatic vessels promote the homing of circulating tumor cells to lymph nodes and their proliferation through lymphangiogenesis and chemokine production in response to signaling molecules secreted by tumor cells and the tumor microenvironment (35). The development of adrenal metastases is believed to be influenced by CC- chemokine receptor 6 (CCR6) and its receptor CC- chemokine ligand 20 (CCL20). The overexpression of CCL20 in normal tissues adjacent to adrenal metastases establishes a microenvironment that enhances the attraction of circulating tumor cells expressing CCR6 to the adrenal gland (36). Tumor cells after invading the brain phenotypically modify local normal cells from an anti-tumor to a pro-tumor phenotype and undergo T-cell immunosuppression to achieve a suitable tumor microenvironment (37–40). The E2F1-mediated inhibitory pathway of WNT5A expression and the CXCL12/CXC- chemokine receptor 4 (CXCR4) chemokine receptor signaling pathway facilitate the invasion of circulating tumor cells into the brain (41).

The microenvironment plays a pivotal role in both tumorigenesis and metastasis. Consequently, targeted therapy directed at microenvironmental signaling pathways may offer a novel perspective for enhancing cancer treatment efficacy.

## 4 Conclusion

We report a case of a female lung cancer patient with metastases to the left atrium and extrathoracic lymph nodes et al. Remarkably, this patient remained asymptomatic from the onset of the disease until the development of metastasis, underscoring the growing importance of regular medical checkups for early disease detection. Furthermore, when the primary cancer lesion is identified, it is crucial to conduct multimodal imaging examinations, including those of the heart. Additionally, adopting intervention therapy from a microenvironmental perspective may offer a novel approach to more effective cancer treatment.

## Data Availability

The raw data supporting the conclusions of this article will be made available by the authors, without undue reservation.
